# Supplementary material for: Hybrid Models and Biological Model Reduction with PyDSTool
Source: PLoS Comput Biol. 2012 Aug 9;8(8):e1002628. doi: 10.1371/journal.pcbi.1002628 (PMC3415397; doi:10.1371/journal.pcbi.1002628)
Supplement: Text S4 — Complete source code for the PyDSTool package (version 0.88.120504). Includes API documentation and help files linking to web pages. This file is identical to the current public release on Sourceforge.net. (ZIP) [file pcbi.1002628.s004.zip › PyDSTool/html/PyDSTool.Toolbox.neuro_data.estimate_spiking-class.html]

xml version="1.0" encoding="ascii"?


PyDSTool.Toolbox.neuro\_data.estimate\_spiking


| Home | Trees | Indices | Help | PyDSTool |
| --- | --- | --- | --- | --- |

Package PyDSTool ::
Package Toolbox ::
Module neuro\_data ::
Class estimate\_spiking

[hide private]

[frames] | no frames]

# Class estimate\_spiking

source code

```
object --+
         |
        estimate_spiking
```

---

Estimate pattern of spiking in tonic or burst patterns.


|  |  |
| --- | --- |
| Instance Methods | [hide private] |

|  |  |
| --- | --- |
| \_\_init\_\_(self, x, t, filt\_coeffs, sense=`'``up``'`)  Pass only 1D pointset. | source code |

|  |  |
| --- | --- |
| eliminate\_group(self, xf, spike\_ixs) | source code |

**Inherited from `object`**:
`__delattr__`,
`__getattribute__`,
`__hash__`,
`__new__`,
`__reduce__`,
`__reduce_ex__`,
`__repr__`,
`__setattr__`,
`__str__`


|  |  |
| --- | --- |
| Properties | [hide private] |

**Inherited from `object`**:
`__class__`


|  |  |
| --- | --- |
| Method Details | [hide private] |

|  |  |
| --- | --- |
| \_\_init\_\_(self, x, t, filt\_coeffs, sense=`'``up``'`)  *(Constructor)* | source code |

Pass only 1D pointset. If spikes are in the "positive"
direction of the variable, use sense='up', else use 'down'.

Overrides: object.\_\_init\_\_

  


| Home | Trees | Indices | Help | PyDSTool |
| --- | --- | --- | --- | --- |

|  |  |
| --- | --- |
| Generated by Epydoc 3.0.1 on Fri May 4 15:24:09 2012 | http://epydoc.sourceforge.net |
